# Artificial Intelligence in Regenerative Medicine: Applications and Implications

**DOI:** 10.3390/biomimetics8050442

**Published:** 2023-09-20

**Authors:** Hamed Nosrati, Masoud Nosrati

**Affiliations:** 1 Biosensor Research Center, Isfahan University of Medical Sciences, Isfahan 81746-73461, Iran; 2 Department of Computer Science, Iowa State University, Ames, IA 50011, USA

**Keywords:** artificial intelligence, regenerative medicine, personalized medicine

## Abstract

The field of regenerative medicine is constantly advancing and aims to repair, regenerate, or substitute impaired or unhealthy tissues and organs using cutting-edge approaches such as stem cell-based therapies, gene therapy, and tissue engineering. Nevertheless, incorporating artificial intelligence (AI) technologies has opened new doors for research in this field. AI refers to the ability of machines to perform tasks that typically require human intelligence in ways such as learning the patterns in the data and applying that to the new data without being explicitly programmed. AI has the potential to improve and accelerate various aspects of regenerative medicine research and development, particularly, although not exclusively, when complex patterns are involved. This review paper provides an overview of AI in the context of regenerative medicine, discusses its potential applications with a focus on personalized medicine, and highlights the challenges and opportunities in this field.

## 1. Introduction

Artificial intelligence (AI) refers to the development of computer systems that can perform tasks that would typically require human intelligence. This includes learning, reasoning, perception, and problem-solving. AI systems are designed to mimic human cognition and to work autonomously, learning from data and prior experiences to improve their performance over time [1,2]. The concept of AI has been around for decades. Still, recent advances in machine learning, deep learning, and natural language processing have made it possible to develop more sophisticated AI systems. Machine learning empowers researchers to analyze vast amounts of data, recognize patterns, make predictions based on that data [3,4], and even learn from their mistakes and adjust their behavior accordingly [5] without being explicitly programmed. Machine learning is used in a wide range of applications, including natural language processing [6], image recognition [7], autonomous vehicles [8], and biomedical engineering [9].

**Figure 1:**
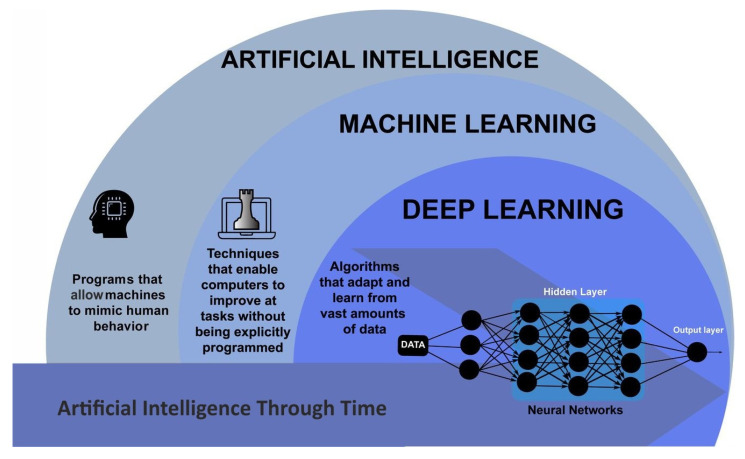
Schematic outlining the AI areas. Adapted from [20].

Deep learning is a subset of machine learning that uses artificial neural networks to learn from data. These neural networks are designed to mimic the structure and function of the human brain, allowing them to identify more complex patterns and make decisions based on the data they have been trained with [10,11]. Deep learning has revolutionized the field of artificial intelligence, enabling machines to perform tasks that were once thought to be impossible. One of the key advantages of deep learning is its ability to handle large and complex datasets [12]. Traditional machine learning algorithms struggle to make sense of data that is too vast or too complex for humans to process. On the other hand, deep learning algorithms can handle millions of data points and identify patterns that would be impossible for a human to detect [13,14]. Another advantage of deep learning is its ability to learn and improve over time [15]. Traditional machine learning algorithms commonly do not offer memory, requiring humans to manually adjust the parameters and settings to improve their performance. Some deep learning algorithms, such as Long Short-Term Memory [16] and Recurrent Neural Networks [17], can adjust themselves automatically based on the data they are processing. This means that deep learning algorithms can continue improving and evolving as they process more data (Figure 1) [18,19].

Regenerative medicine is a rapidly evolving field that seeks to restore or replace damaged or diseased tissues and organs through advanced technologies such as stem cell-based therapies, gene therapy, and tissue engineering [21,22]. With the potential to revolutionize medical treatment, regenerative medicine offers hope for patients suffering from a wide range of conditions, including heart disease, diabetes, and neurological disorders [23,24]. However, developing effective regenerative therapies requires the ability to analyze large amounts of complex data, which is where AI comes in.

This paper offers a distinct contribution by synthesizing and analyzing the available literature on AIs applications in regenerative medicine, providing an overview, identifying gaps in the existing literature, and proposing novel research directions. By adopting a holistic perspective, we not only consider empirical studies but also include theoretical perspectives and expert opinions. This approach broadens the scope of our analysis and allows for a more comprehensive understanding of the topic. By incorporating diverse sources of evidence, our manuscript offers a unique perspective that is not limited to a single methodological approach. Thus, our study presents a novel synthesis of the literature, shedding light on the potential of AI to revolutionize regenerative medicine.

## 2. AI in Regenerative Medicine

AI has become a crucial aspect in performing computational simulations and in silico studies in medical applications and offers several advantages, such as lower costs and faster results compared to other medical investigation approaches, such as clinical and laboratory methods [25,26,27]. Currently, multiple ongoing initiatives are aimed at incorporating AI into a wide range of fields, including but not limited to medicine, pharmaceuticals, and healthcare [28,29,30]. These projects aim to leverage the power of AI to enhance and streamline various processes, such as drug development, disease diagnosis, and medical treatment. By integrating AI, researchers and practitioners hope to achieve more accurate and efficient outcomes, ultimately improving the quality of life for individuals and communities [30,31]. To be more specific, deep learning can help accelerate the development of regenerative therapies by facilitating tasks such as analyzing large datasets of molecular and genetic data and identifying patterns and correlations that may be missed by human researchers. This can help researchers better understand the underlying disease mechanisms and develop more effective therapies to address them. Some of the most important scopes of regenerative medicine for which AI could be useful are discussed in this section.

### 2.1. Drug Discovery

There are a huge number of molecules in the chemical space, presenting both opportunities and challenges in drug discovery and development. In the context of regenerative medicine, drug discovery involves identifying molecules, biologics, or other therapeutic agents that can promote tissue regeneration and functional recovery. The development of drugs is limited by the lack of advanced technologies. Traditional drug development processes can be time-consuming and expensive, as they involve synthesizing and testing a large number of compounds to identify potential drug candidates. Another major concern in drug discovery is ensuring that the potential drug candidates are safe and effective [32]. To overcome these challenges, AI has emerged as a powerful tool that can analyze large datasets of chemical compounds to predict which treatments work best for certain illnesses. It has become possible to detect patterns and associations by analyzing chemical structures and properties, which can help identify potential drug candidates. This information can be used to prioritize compounds for further testing and development. AI can also assist in validating the drug target, which is the specific biological molecule or pathway a drug aims to interact with. By using AI, researchers can gain insights into the drug target’s function and potential effectiveness, saving time and resources. Additionally, it can predict the toxicity of potential drug candidates by analyzing their chemical structures and properties. This can help to identify potential safety concerns early in the drug discovery process, reducing the risk of adverse events. Moreover, AI can assist in designing new molecules that are optimized for specific therapeutic applications. Moreover, it can facilitate the identification of new molecules that are more likely to be effective treatments for particular diseases. While AI has the potential to enhance the drug discovery process significantly, researchers and clinicians must address challenges related to data quality, transparency, and regulatory issues. By addressing these challenges, they can continue to refine AI technologies and improve the efficiency and effectiveness of drug discovery. There are currently various AI tools used in different aspects of drug discovery and development, including drug design (e.g., target protein structure prediction, drug-protein interactions, and *de novo* drug design) and drug screening (e.g., prediction of physicochemical properties, bioactivity, and toxicity) [33,34]. Some of these tools are presented in Table 1.

### 2.2. Disease Modeling

Disease modeling involves creating in vitro models of diseases, which can be used to study the underlying mechanisms of the disease and test potential treatments. By employing disease modeling, researchers can gain a comprehensive understanding of disease pathology, identify new therapeutic targets, and gain insights into regenerative processes for restoring normal tissue function. Additionally, disease modeling can also be used to screen potential drugs and identify the most promising candidates for further development [43,44,45]. AI can help researchers analyze data generated from disease models and identify patterns and correlations that may not be immediately apparent. This can help identify new therapeutic targets and potential drug candidates for further development.

One of the key advantages of disease modeling is the ability to create personalized models of diseases using patient-specific cells. This allows researchers to study the disease in a more accurate and relevant context, as each model reflects the unique genetic and environmental factors that contribute to the condition in the patient [46,47]. AI can help identify biomarkers, genetic mutations, and other factors that contribute to the development and progression of diseases. This information can then be used to create more accurate disease models that can be used to identify potential treatments. Furthermore, these models can be used to test the efficacy of personalized treatments, such as gene- or cell-based therapies, which can be tailored to the individual patient’s needs. AI algorithms can be used to identify genetic variations that are associated with specific diseases, allowing researchers to develop personalized treatments based on an individual’s genetic profile. AI could also be considered in the development of gene therapies for rare genetic disorders.

### 2.3. Predictive Modeling

Predictive modeling involves using data to train machine/deep learning models to predict future outcomes based on unforeseen data. Its connection to regenerative medicine is rooted in their mutual goal of advancing personalized medicine and optimizing treatment strategies. Predictive modeling plays a crucial role by providing insights into diverse areas, such as predicting disease progression, identifying patients at risk of developing certain conditions, and optimizing treatment plans. Predictive modeling is a challenging task in healthcare due to the complexity of healthcare data and the large amounts of data involved [48,49]. AI offers high-accuracy predictive models for analyzing clinical and biological data to identify patterns and associations that can be used to predict future outcomes. Machine learning algorithms can identify factors that contribute to the development and progression of diseases. This information can then be used to create more accurate predictive models to identify patients at risk of developing certain conditions and optimize treatment plans. Additionally, AI allows the development of personalized predictive models by analyzing patient data, such as genomics, proteomics, and metabolomics. It helps identify individual disease process differences that can be used to create personalized predictive models. This information is then used to develop personalized treatment plans tailored to individual patients’ specific needs. Furthermore, AI can identify possibilities for creating new medicines by studying biological data. It can uncover targets and paths linked to particular diseases, enabling the development of drugs that aim for these paths and enhance the effectiveness of existing ones.

### 2.4. Personalized Medicine

Personalized medicine aims to provide tailored medical treatments to individual patients based on their genetic, environmental, and lifestyle factors. However, accurately predicting a patient’s response to a particular treatment remains a significant challenge due to the system’s complexity [50,51]. AI can help overcome this challenge by analyzing patient information and identifying patterns and associations that can predict treatment outcomes. One way AI can assist in personalized medicine is by analyzing a patient’s genomic data. AI algorithms can identify genetic variations linked to specific diseases or treatment responses, enabling the development of personalized treatment plans based on the patient’s genetic profile. Another way AI can help is by analyzing patient health data, including electronic medical records, imaging data, and patient-reported outcomes. This data can reveal patterns and associations that predict treatment outcomes and inform personalized treatment plans. For instance, AI algorithms can identify patients most likely to benefit from a specific treatment or predict which patients may experience adverse reactions to a treatment. AI can also develop personalized treatment plans based on patient preferences and values. Analyzing patient-reported outcomes and other data can identify treatment options that align with the patient’s values and preferences. AI has the potential to enhance the effectiveness of personalized medicine significantly, providing new tools and insights for clinicians and researchers. However, issues related to data privacy, bias, and regulatory challenges still must be addressed. By working to overcome these challenges, researchers and clinicians can refine AI technologies and improve patient care quality.

### 2.5. Tissue Engineering

Tissue engineering is an interdisciplinary field that integrates principles of engineering, biology, and medicine to develop novel approaches to repair, replace, or regenerate tissues and organs. This field has emerged as a promising alternative to traditional approaches [52]. However, it faces significant challenges, as summarized in Table 2.

To tackle these challenges, AI has emerged as a powerful tool that analyzes the physicochemical and biological properties of a wide range of materials to predict the most successful outcomes. AI algorithms can identify patterns and associations in cellular behavior and interactions, thereby enabling the prediction of cell behavior in different environments. This information is crucial in designing and optimizing tissue engineering strategies to develop functional organs and tissues.

Scaffolds are one of the key components of tissue engineering, as they provide a structure for cells to grow and form new tissue. The success of tissue engineering approaches depends largely on their ability to create effective scaffolds that can support the growth and differentiation of cells into functional tissue [53]. Scaffolds can be made from a variety of materials, such as ceramics, synthetic polymers, and natural biopolymers, and can be designed to mimic the properties of natural tissue [54]. AI can optimize material properties for specific applications by analyzing their properties and interactions with biological systems. This information can then be utilized to design and develop scaffolds for specific tissue engineering applications. Scaffolds can be fabricated using a variety of techniques, depending on the type of material being used and the desired properties of the scaffold [55]. AI can play a significant role in choosing an efficient and effective scaffold fabrication method for the intended application. AI algorithms can analyze large amounts of data on different materials and fabrication techniques to identify suitable combinations for a specific tissue engineering application. These algorithms can also simulate the fabrication process and predict the properties of the resulting scaffold, which can help researchers optimize the design and reduce the time and cost of the fabrication process. Additionally, AI can assist in quality control by monitoring the fabrication process in real-time and detecting any deviations from the desired parameters. This can help in ensuring that the scaffold is fabricated according to the desired specifications and quality.

### 2.6. Cell Therapy

Cell therapy is a promising field in regenerative medicine that involves the use of living cells to replace or repair damaged or diseased tissues and organs. It is based on the concept that cells have the ability to regenerate and differentiate, which makes them ideal candidates for repairing damaged tissues and organs [56]. Cell therapy can potentially revolutionize the treatment of many chronic diseases and injuries that currently have limited or no treatment options [57,58]. One of the most promising areas of cell therapy is the use of stem cells. Stem cells are undifferentiated cells that have the ability to differentiate into different cell types [59]. They can be obtained from various sources, including embryonic tissue, adult tissue, and umbilical cord blood [60]. While cell therapy has shown promising results in clinical trials, it still faces significant challenges in identifying suitable cells, ensuring their safety, and optimizing their effectiveness [61,62]. This is where AI comes in. AI has the potential to revolutionize cell therapy by enabling researchers to analyze vast amounts of data and develop new insights into how cells work. One of the key benefits of using AI in cell therapy is its ability to help identify the best cells for a particular patient. By analyzing a patient’s genetic information and medical history, AI algorithms can predict which cells will most likely be effective in treating their condition. AI can also help researchers identify the optimal conditions for growing cells. In cell therapy, the delivery of cells to the target site is a critical step that can significantly impact the success of the treatment. AI can help improve the delivery of cells by optimizing the route of administration and ensuring the cells reach the target site effectively. AI can also help determine the optimal dose and timing of cell delivery to maximize therapeutic benefits. Additionally, it can assist in tracking the cells after delivery, monitoring their migration and survival, and detecting any adverse effects. This can aid in adjusting the treatment plan and improving patient outcomes. Despite its potential benefits, there are also limitations to the use of AI in cell therapy. One major limitation is the quality and quantity of available data. AI algorithms require large amounts of high-quality data to accurately predict outcomes. However, in the field of cell therapy, patient data are often limited and heterogeneous, making it challenging to train AI models effectively. AI models are only as good as the data they are trained on, and there may be biases or inconsistencies in the data that can affect the accuracy of AI predictions. Another limitation is the complexity of biological systems. Cell therapy involves excessively intricate interactions between cells and tissues, making the analysis difficult for many of the machine and deep learning algorithms to model them accurately.

### 2.7. Clinical Trial Design

Clinical trial design plays a crucial role in the field of regenerative medicine, as it enables the evaluation of drugs and novel regenerative therapies in terms of their safety and efficacy. Nonetheless, designing clinical trials can be convoluted and time-consuming, with multiple variables to consider, including patient selection, study endpoints, and statistical analysis [63,64]. In this context, AI has emerged as a powerful tool to address these challenges and enhance the accuracy and efficiency of clinical trial design. AI can assist in clinical trials by identifying patients most likely to respond to new treatments. By analyzing datasets of clinical and biological data, AI algorithms can identify biomarkers, genetic mutations, and other factors associated with treatment response, leading to the identification of patient populations that are most likely to benefit from new treatments. This reduces the number of patients needed to achieve statistically significant results, improving the efficiency of clinical trials. AI can also improve the selection of study endpoints by analyzing datasets of clinical trial data to identify endpoints that are more sensitive and specific than traditional endpoints. This ensures clinical trials measure clinically relevant outcomes and provide more meaningful results. Additionally, AI can improve the statistical analysis of clinical trial data by using machine learning algorithms to analyze and interpret complex datasets. This can help to identify patterns and insights that may not be immediately apparent to human analysts, improving the accuracy and efficiency of statistical analysis.

### 2.8. Patient Monitoring

Patient monitoring is not only essential for assessing the effectiveness, safety, and progress of treatments but also crucial for the identification and management of potential complications. This ensures optimal outcomes through timely interventions and optimized treatment outcomes [65,66]. However, patient monitoring can be complex and time-consuming due to the large amounts of data that must be analyzed and interpreted. This is where AI can significantly help by analyzing large datasets of patient data to identify patterns and anomalies that may indicate a change in patient health. By using machine learning algorithms to analyze data from wearable devices, electronic health records, and other sources, AI can identify changes in patient health that may not be immediately apparent to healthcare providers. This information can then be used to alert healthcare providers to potential problems and enable them to take proactive measures to prevent complications by using AI-generated solutions. Additionally, AI can provide real-time insights into patient health by using natural language processing and other AI technologies. For example, AI algorithms can analyze patient data to identify trends and patterns indicating a need for medication adjustments, lifestyle changes, or other interventions. AI can also improve the accuracy and efficiency of patient monitoring by automating routine tasks such as data entry and analysis, enabling healthcare providers to focus on more complex tasks and enhance the quality of patient care. Furthermore, AI can reduce the time and cost associated with patient monitoring by enabling healthcare providers to monitor more patients simultaneously and identify potential problems earlier.

### 2.9. Patient Education

Patient education is essential to healthcare, as it enables patients to be actively involved in their health and make informed decisions [67]. However, patient education can be challenging due to the diverse backgrounds, preferences, and levels of health literacy among patients [68]. AI can improve patient education by addressing these challenges. Generative language models such as ChatGPT [69] can help by providing personalized education materials tailored to individual patients’ specific needs and preferences. In this regard, AI algorithms can identify differences in education needs and preferences and generate personalized education materials such as videos, infographics, and interactive tools. AI technologies can also improve the accessibility and usability of educational materials by using natural language processing to present materials in plain language in visually appealing and engaging ways. AI can also identify gaps in patient education and improve education interventions. By analyzing patient outcomes and behavior data, AI algorithms can provide insights into improving education interventions and identify areas where education is lacking or ineffective. Therefore, AI has the potential to improve the effectiveness and efficiency of patient education significantly. However, challenges such as data privacy, ethics, and trust need to be addressed. Researchers and healthcare providers need to work together to refine AI technologies and ensure they are used ethically and in a way that builds patient trust.

### 2.10. Regulatory Compliance

Regulatory compliance refers to ensuring that an organization or individual complies with the laws, regulations, and standards that apply to their industry or field. Regulatory compliance is particularly crucial in the complex and rapidly evolving field of regenerative medicine. In this regard, AI can improve data collection and analysis by utilizing machine learning algorithms to identify patterns and insights that may be difficult for human analysts to detect. This information can then be used to ensure products and therapies comply with regulatory standards. Additionally, the transparency and traceability of data and processes can be enhanced through blockchain technology and AI-powered tools. This enables tracking the entire lifecycle of products or therapies, from development to patient outcomes, ensuring transparency and the availability of relevant data for analysis and review. Furthermore, personalized treatments can be developed by using AI algorithms to tailor treatments to the specific needs and characteristics of individual patients. This reduces the risk of adverse events and ensures compliance with regulatory standards. While AI has the potential to significantly improve regulatory compliance in regenerative medicine, challenges such as data privacy, ethics, and regulatory oversight need to be addressed. By addressing these challenges, researchers and clinicians can continue to refine and develop AI technologies to enhance the safety and efficacy of products and therapies.

## 3. AI in Other Fields Related to Regenerative Medicine

### 3.1. Immunotherapy

Immunotherapy is a treatment that harnesses the body’s immune system to specifically target cancer cells [70]. It involves administering drugs that work by blocking or stimulating specific immune cell receptors [71] or introducing modified immune cells that can recognize and attack cancer cells [72]. This approach has the potential to create a long-lasting anti-tumor response in specific cancer patients. Immunotherapy is a rapidly evolving field, and ongoing research is focused on identifying new targets and developing more effective therapies to improve the results. The efficacy of current cancer immunotherapy treatments relies on agents that stimulate or enhance the immune system’s response to cancer. In addition to the successful use of immune checkpoint inhibitors, neoantigen vaccinations, and T-cell transfer, there is hope for further advancements utilizing innovative technologies and methods [73].

The primary goal of immunotherapy is to customize treatments based on a patient’s unique disease characteristics, specifically related to the immune system’s response to tumor cells. Researchers are dedicating their efforts to identifying predictive biomarkers of both response and resistance to treatment and creating treatment models. To better understand the interactions between the immune system and tumors, they study the dynamics of each cell population involved. By utilizing simulation models with differential equations for each cellular subtype and chemical mediator during immune interactions, researchers can monitor changes in specific cell populations over time. Additionally, these mathematical models can provide insight into how various immune cells respond to tumors with different immunogenicity and growth rates [73,74].

With the emergence of AI, simulation models have been updated to include more complex aspects of the tumor-immune relationship. This includes accounting for the spatial dynamics of tumors, cellular heterogeneity, and cytokine activity, as well as other signaling and modulating factors. To accurately model immunogenic tumors, researchers must consider the heterogeneous spatial distribution of both tumor and immune cell populations. The impact of cytokines, which mediate tumor-immune interactions, adds an additional level of complexity to these models. This may require accounting for reaction or diffusion processes, such as exchanging substances with the local microenvironment.

The process of translating immunotherapy concepts into clinical practice can be lengthy and challenging. To address this, researchers have increasingly turned to AI models that can predict hypothetical treatment outcomes and provide insights into the underlying mechanisms that determine the success or failure of immune therapies. Personalized mathematical models have also been developed to enhance the efficacy of newly developed immunotherapies during clinical trials and to increase their chances of regulatory approval. However, this personalized approach does not align with the current model of therapy development, which involves applying pre-determined treatment schedules uniformly to all patients in trial arms. Implementing personalized models would require a shift in the current paradigm of clinical trials.

To improve the specificity and accuracy of models used in clinical practice, more detailed and spatially resolved clinical data are required. The availability of such data will allow researchers to develop more accurate models of specific cancers and treatments by incorporating detailed characteristics of tumor-immune interactions. Collaboration between AI specialists and clinicians is necessary to facilitate the development of well-informed clinical trials. This collaboration would allow qualitative hypotheses to be quantified, leading to the personalization and optimization of doses and the scheduling of immunotherapeutic protocols. Such an approach would streamline the transition from innovative concepts to clinical practice and ultimately improve clinical outcomes for individual patients [73].

### 3.2. Genetic Engineering

Genetic engineering is a process that involves altering the genetic material of an organism in order to modify or enhance its characteristics. This technology allows for the targeted manipulation of specific genes within an organism’s genome, which can result in desired changes in traits or functionalities. The process may include the insertion, deletion, or modification of genes to achieve the desired outcomes [75,76]. Genetic engineering plays a significant role in regenerative medicine. One of the key applications of genetic engineering is to modify stem cells to enhance their properties and direct their differentiation into specific cell types, leading to advancements in their regenerative potential [77]. Genetic engineering can also be used to modify the genetic material of cells in the body to enhance their therapeutic activity [78]. Genetic modifications can be employed to produce induced pluripotent stem cells (iPSCs), which are adult cells that have been reprogrammed. These cells can then be differentiated into various cell types and used for biomedical applications, including transplantation and drug testing [79]. Furthermore, genetic engineering is also used in the development of gene therapies, which involve introducing new genes into the body to treat or prevent diseases. Gene therapies can replace or repair defective genes that cause genetic disorders or introduce new genes that can enhance the body’s natural regenerative abilities [80,81].

In genetic engineering, AI has two primary functions: (1) detecting harmful genes and (2) finding appropriate treatments for genetic diseases. Analyzing the massive amount of data found in an individual’s DNA is a very laborious and time-consuming task for humans. However, machines can be utilized to efficiently and precisely perform this analysis, fulfilling their primary purpose of lessening the burden of tedious tasks. AI algorithms can be employed to compare gene expression levels in malignant and normal tissue samples of a cancer patient, enabling predictions to be made about any mutated genes in the patient’s DNA. The algorithms would use the frequency of gene expression in malignant and normal samples to train and make predictions, continuously incorporating new data to refine the accuracy of their predictions. Three-dimension imaging is being leveraged by AI to identify genetic mutations in tumors. By utilizing deep learning and neural networks, machines can accurately detect the presence of a mutation, enabling doctors to devise better treatment plans for patients without the need for biopsy tissue samples or surgical procedures. These developments in machine learning have promising potential for streamlining disease diagnosis, particularly for cancer.

We are entering a new age where genome-editing tools will allow us to inactivate or correct genes that cause diseases. This breakthrough offers the possibility of life-saving treatments for individuals suffering from genetic disorders [82]. Despite significant advancements in technologies such as CRISPR, the risk of errors remains high, and safety must be prioritized for gene editing to advance. Machine learning algorithms can aid in identifying where the alteration should be made and how to ensure proper repair of the DNA strand, thus mitigating the potential for errors throughout the gene editing process.

As mentioned earlier, AI is particularly beneficial in personalized medicine. Our unique DNA has a vast number of variations when compared to others, indicating that cancer-causing mutations in one individual’s genome will differ in location and level from those in another with the same disease. AI can pinpoint which genes have been affected by harmful mutations, enabling them to be targeted in gene therapy.

While AI can reduce technical errors in gene editing and improve safety, it raises several ethical questions. Some argue that using AI may increase the risk of malfunctions and that the non-human aspect of AI may be more harmful than beneficial. The introduction of AI into genetic engineering also raises concerns about unequal access to gene therapy based on wealth and the potential misuse of genome editing for non-healthcare purposes, such as physical enhancement. Additionally, religious and moral objections must be considered when contemplating genome editing as a possible treatment for genetic diseases. It is important to note that the accuracy and impartiality of machine learning algorithms and tools depend on the quality of the data they are fed and the nature of the algorithms. Despite the advancements in AI technology, the machine is incapable of independent thinking and is only as good as the information it is provided with.

### 3.3. Nanobiotechnology

Nanobiotechnology is a field of science that combines nanotechnology and biology [83]. It involves studying and manipulating biological systems at the nanoscale, typically between 1 and 100 nm in size [84]. Nanobiotechnology aims to develop new materials, devices, and systems to address diverse challenges in various applications. Researchers can create nanomaterials with specific properties that can interact with biological systems in unique ways. For example, nanoparticles can be engineered to bind to specific cells or molecules, allowing for highly targeted drug delivery or sensing [85,86]. Another important aspect of nanobiotechnology is the ability to study biological systems at the nanoscale. Researchers can use advanced imaging techniques to visualize and analyze biological structures and processes at the molecular level [87]. This can lead to a better understanding of biological systems and the development of new therapies and treatments.

Nanobiotechnology can help drug delivery by developing nanoparticles that can be used to deliver drugs directly to specific cells or tissues, improving efficacy and reducing the side effects of drugs [88]. The unique properties of nanoparticles, such as their small size and surface area-to-volume ratio, allow them to interact with biological systems in different ways [89]. Additionally, nanoparticles can be designed to release drugs in a sustained or controlled manner, improving the drug’s pharmacokinetics and reducing toxicity [90,91]. Furthermore, nanoparticles can also protect drugs from degradation and clearance by the immune system, increasing their bioavailability and improving their therapeutic effect [92,93]. AI can be used to identify the most promising nanoparticles for drug delivery based on their biological and chemical properties. It can also help optimize the design of nanoparticles to enhance their stability, drug-loading capacity, and targeting efficiency. Additionally, AI can be used to predict the pharmacokinetics and toxicity of nanoparticles, allowing researchers to optimize their properties for safe and effective drug delivery. Moreover, AI can improve drug delivery by enabling the development of smart nanoparticles that can respond to specific stimuli in the body, such as changes in temperature or pH. These smart nanoparticles can be designed to release drugs only when they reach the target tissue, reducing side effects and improving drug efficacy. AI has the potential to significantly enhance the development of safer and more efficient therapies for a wide range of diseases.

Advances in nanotechnology have remarkably impacted tissue engineering, as numerous nanotechnology-based approaches have been developed to address a wide range of issues. For instance, nanomaterials could be incorporated into scaffolds to enhance their mechanical properties. This could provide mechanical support for the tissue during regeneration [94]. Nanomaterials can be functionalized with bioactive molecules, enhancing the scaffold’s ability to interact with cells and promote tissue regeneration [95]. Nanomaterials can be designed to mimic the structure and function of a natural extracellular matrix, providing a more biomimetic environment for cells to grow and differentiate, leading to better tissue regeneration [96,97]. AI algorithms can be used to analyze data on the mechanical, chemical, and biological properties of different nanomaterials and polymers and predict how they will interact with cells and tissues. This can enable researchers to design scaffolds with optimal properties and functions, such as enhanced mechanical strength, improved biocompatibility, and better cell adhesion. For example, AI can be used to predict the optimal composition and ratio of different materials in the scaffold to achieve the desired mechanical properties. In addition to optimizing the design of the scaffold, AI can also be used to optimize the fabrication process of nanocomposite scaffolds by predicting the optimal processing parameters for the synthesis of nanocomposite scaffolds. This can lead to the development of scaffolds with uniform and controlled properties, which is crucial for their successful application in tissue engineering. Furthermore, AI can also be used to predict the performance of nanocomposite scaffolds in vivo by simulating the interaction of the scaffold with biological systems, such as the immune system and surrounding tissues. This can help predict the scaffold’s long-term performance, including its biocompatibility, degradation rate, and ability to support tissue regeneration.

The main challenges faced when incorporating AI into nanobiotechnology-based therapies are the lack of sufficient high-quality data, the complex interactions involved in biological systems, and model interpretability. Compiling and annotating large datasets on nanomaterials’ biological, chemical, and physical properties is a major challenge for training accurate AI models. Hence, the lack of proper data might cause bias in the model results. Biological systems are inherently complex, with multi-factorial interactions dependent on parameters such as nanomaterials’ size, shape, and surface properties. Modeling these complex size- and shape-dependent interactions between nanomaterials and biological entities using AI is challenging since many of the simpler models are not able to find patterns in the data accurately, and using more complex models requires more computational resources. Additionally, the behavior of nanomaterials within living biological systems introduces further complexity and variability. Factors such as immune response and natural variation between individuals result in highly variable in vivo performance of nanomaterials. This makes it challenging to precisely predict and optimize their behavior inside the body using AI. However, these challenges are actively being addressed. Researchers are extensively characterizing nanomaterials through experiments to develop extensive property datasets required for training sophisticated AI models. Efforts are ongoing to design multi-factor AI algorithms, such as Convolutional Neural Networks, that can capture the complexity introduced by parameters such as size and shape during biological interactions.

### 3.4. Microfluidics

Microfluidics is a field of research that studies the behavior, manipulation, and control of fluids and particles at the microscale level [98]. It involves the study of fluid behavior, transport, and interactions at the microscale level and has applications in a wide range of fields, such as biotechnology [99], chemical synthesis [100], environmental monitoring [101], and diagnostics [102]. Microfluidic devices are small-scale systems that manipulate and control the flow of fluids at the micrometer scale. They are typically made up of channels, chambers, and valves that can be designed to perform various tasks, such as mixing, separating, and analyzing fluids [103,104]. Microfluidic devices are often used to perform complex chemical and biological assays, where precise control over the flow of fluids is essential for accurate and reliable results [105,106]. Microfluidic technology has the potential to significantly advance the field of regenerative medicine by enabling the precise control of cellular microenvironments and the development of complex tissue structures [107]. Microfluidic devices can control stem cell culture conditions, enabling their differentiation into specific lineages for tissue engineering applications. These devices can mimic in vivo environments to develop complex structures by controlling flow velocity, nutrient concentration, and other environmental factors [108,109,110]. They can also be used to sort and isolate specific cell populations, which are then used for cell therapy applications [111]. Microfluidics can also be used for drug screening, where different compounds can be tested for their effectiveness in promoting tissue regeneration. By exposing cells or tissues to various drugs in a controlled manner, researchers can identify the most effective drugs for tissue regeneration [112]. Microfluidic devices can be used to analyze small volumes of biological fluids such as blood, saliva, and urine, enabling the identification of specific biomarkers associated with various diseases [113,114]. Analyzing these biomarkers rapidly and cost-effectively can allow the development of personalized treatments, which can be used to identify diseases at an early stage and monitor disease progression. By precisely controlling drug delivery to specific cells or tissues, this technology can significantly enhance the effectiveness of treatments and reduce side effects, leading to better patient outcomes [115,116,117].

There are several technical challenges in microfluidics, including scaling up device fabrication, device operation/sample processing, and the programmability of chips [118]. Currently, many devices are made from polydimethylsiloxane (PDMS) using soft lithography [119,120]; however, PDMS has limitations such as swelling in many non-polar organic solvents [121] and leaching molecules [122,123]. Identifying affordable, scalable materials for laboratory and mass production is challenging. Potential materials include organic materials (e.g., elastomers, thermosets, plastics, hydrogels, and paper), silicon, and glass [124,125]. Device operation often relies on external equipment. Integrating smaller, microfluidic-scale equivalents of this equipment could make devices more accessible and affordable [118,126]. While microfluidic chips do not have the programmability of microprocessors, connecting discrete function-specific chips into a single system is difficult due to incompatible specifications around flows, geometry, and actuation [118,127]. Improving chip design for compatibility in more extensive systems is an area for progress. Addressing these materials, fabrication, operation, and integration challenges would help scale up microfluidics.

AI has great potential to facilitate working on many of the technical challenges faced in microfluidics. One way is through material identification and selection. AI algorithms can analyze vast datasets on material properties and performance under different conditions to help screen and identify new affordable and scalable materials suitable for microfluidics fabrication and operation. This will help tackle one of the key challenges around identifying compatible materials. AI can also optimize microfluidics fabrication processes by finding proper combinations of parameters, materials, and techniques through simulations and data analysis. This will improve the yield, throughput, and reproducibility of the fabrication processes. AI tools can further assist in integrated device and system design through modeling and simulation. They can help in designing microfluidic chips, modules, and systems that have better compatibility and integration of functional components and are more compact in size. Automating tasks such as sample loading, running experiments, and data collection through computer vision and AI-based controls can make microfluidic systems simpler to operate with minimal human intervention. Furthermore, microfluidic devices can generate vast amounts of data, including information on cell behavior, fluid dynamics, and chemical reactions. However, this data can be challenging to analyze and interpret, especially when dealing with large data sets composed of numerous parameters. AI algorithms can be trained to analyze this data, identify patterns and trends, and make predictions based on this information. This can significantly enhance the efficiency and effectiveness of data analysis, leading to better outcomes in research and development.

## 4. Considerations for AI Applications in Regenerative Medicine

Although AI has offered many advantages to regenerative medicine and opened up new research opportunities, it is also accompanied by certain considerations and challenges. The notable ones are listed below.

### 4.1. Trustworthiness

**Figure 2:**
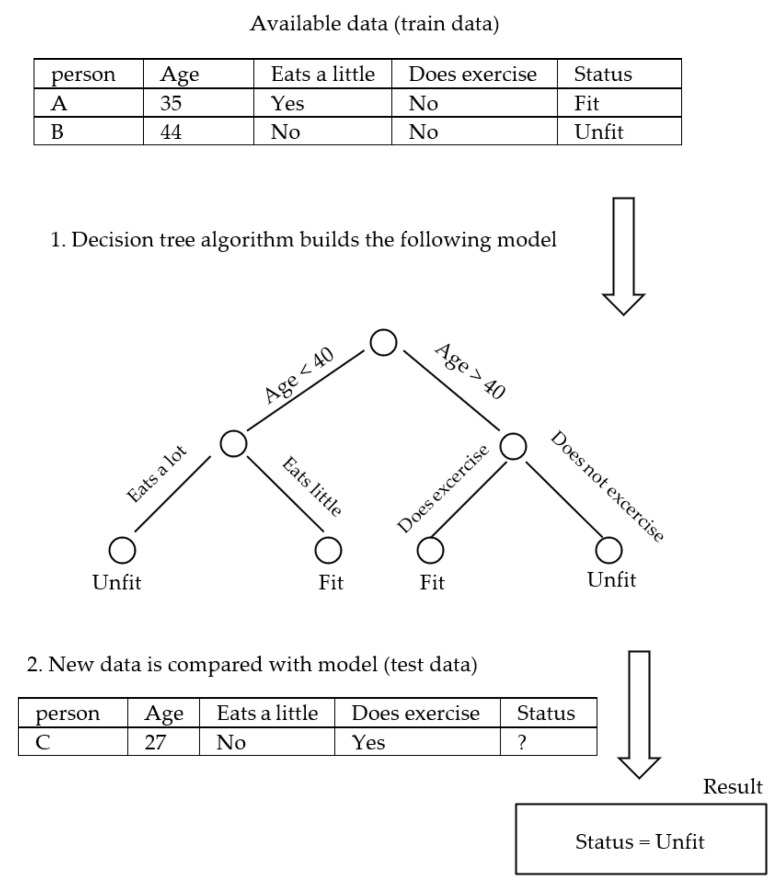
Decision tree (interpretable).

**Figure 3:**
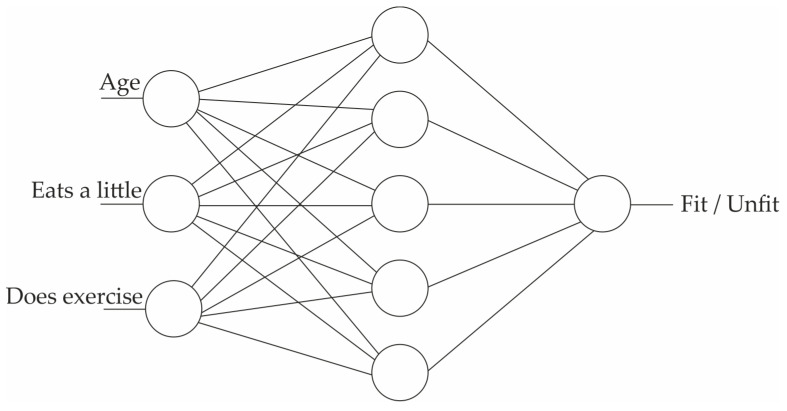
Multi-Layer Perceptron (not interpretable).

Probably the most important consideration in medicine is trustworthiness [128], which refers to the validity and reliability of a model. The trustworthiness of AI is closely related to the interpretability of the model and deals with the answer to this question: “How can people trust the AI-generated information when the result is not interpretable?” An example of an interpretable model is a decision tree [129], shown in Figure 2. The top node of the tree represents the entire dataset. It is the starting point for the decision-making process. Internal nodes represent features or attributes from the dataset. Each internal node makes a decision based on a specific feature. The branches that connect nodes represent the decision outcomes or choices based on the feature’s values. Finally, at the lowest level, the leaf nodes are the endpoints of the decision tree. They provide the final output, which can be a class label (for classification problems) or a numerical value (for regression problems). A decision tree algorithm builds such a tree based on the existing data. Once the tree is created, the unforeseen data could be checked against it and decided for the category or the value that should be assigned. In the example of Figure 2, the decision tree algorithm gets the available data of persons A and B and builds the model tree, which can identify the status of unforeseen person C. In terms of interpretability, the decision tree identifies person C as unfit, and the reason for such a classification is that “age < 40” and “eats a lot”. However, this is not the case with Figure 3, where a Multi-Layer Perceptron Neural Network is shown [130]. An MLP consists of many small computational units with an activation function that determines their output. To use the MLP, it should first be trained with the available data. Each data record runs through the network, and each neuron tunes itself so that the network’s final output converges to the right answer. At the end of the training phase, the MLP would be a collection of tuned neurons that were able to classify the unforeseen data. However, in terms of interpretability, it is usually impossible to look at an MLP and understand why it might label a person as fit or unfit since the network might have thousands of neurons organized in several layers. Indeed, many machine learning and deep learning models are like a black box, containing such a large amount of information that they are excessively difficult, if not impossible, to interpret.

Some ways to address the interpretability issue include developing simplified models using techniques such as knowledge distillation [131]. Such models retain high performance while also being more interpretable. Another method uses algorithms such as SHAP (SHapley Additive exPlanations) values [132]. These values can indicate the impact of each feature on a model’s prediction, making it easier to understand how different features contribute to the overall result. Additionally, researchers are creating visuals such as Confusion Matrices and Calibration Plots that help scientists explore model decisions. These tools provide visualization of feature importance and relationships, allowing for a better understanding of the factors influencing the model’s output.

Another consideration relevant to trustworthiness is data quality. High-quality and diverse datasets are essential for training effective AI models. Many of the models can inherit biases present in the data used for training. Hence, the result of the model would be negatively affected. Ensuring fairness and mitigating bias in AI algorithms is a significant challenge to avoiding discrimination in decision-making. In some areas of regenerative medicine, obtaining clean and well-distributed data might be difficult, and consequently, the AI results might become invalid.

### 4.2. Model Application

Not all the models are appropriate for all problems. Selecting a model depends on factors such as the problem’s nature, the data’s size and quality, interpretability requirements, computational resources, and so on. A problem might be of the classification, regression, or clustering type. Moreover, in a different taxonomy, either of the following types of models might be appropriate for a problem:

- Supervised models: the algorithms learn from labeled data, where the input data are paired with corresponding target or output values. The goal is to predict these target values for new, unseen data. A few examples of supervised models are linear [133] and logistic regressions [134], decision trees [129], random forests [135], support vector machines [136], Convolutional Neural Networks [137], and Recurrent Neural Networks [138]. Some examples, such as linear regression, could be used for classification (finding the category of the data) and regression (finding the numerical value of the data), and some are specific to classification or regression.
- Unsupervised models: the algorithms work with unlabeled data, seeking to discover patterns, structures, or relationships within the data without explicit guidance on what to look for. They are also known as clustering algorithms. A few examples of unsupervised models are K-Means [139], Hierarchical Clustering [140], and Generative Adversarial Networks [141].
- Reinforcement learning: an agent learns to make decisions by interacting with an environment. It receives feedback in the form of rewards or penalties based on its actions to maximize cumulative rewards over time. The most well-known example of this category is Q-Learning [142], which is used in tasks such as tic-tac-toe game playing and simple robot control.

Moreover, a specific model is not guaranteed to be better than others. Choosing a model depends on various parameters, as mentioned before. However, algorithm selection is often an iterative process that needs refining as more insights from experiments and data analysis are obtained.

### 4.3. Multidisciplinary Collaboration

Building an AI system necessitates a solid foundation of technical knowledge. AI, with its complex algorithms, intricate neural networks, and extensive data manipulation, demands a deep understanding of computer science, mathematics, and programming languages. Proficiency in machine learning frameworks, such as TensorFlow [143] or PyTorch [144], and expertise in data preprocessing, feature engineering, and model selection are vital. Additionally, a grasp of software engineering principles is crucial for developing scalable, efficient, and maintainable AI solutions. Demanding a broad range of technical expertise promoted multidisciplinary collaboration. A real-world example of such collaboration is Japan’s national strategy for developing AI technology in the medical field. An early challenge for Japan was having relatively few AI experts compared to countries such as the US and China. To address talent shortages, Japan launched the AI Technology Strategy Council in 2016 to make AI a focus of its Society 5.0 national strategy. The strategy emphasizes using AI to boost productivity, healthcare, and mobility. Japan aims to capitalize on aggregating its population of 125 million citizens’ health data through laws and infrastructure to create one of the largest centralized medical data repositories. The government and private sector in Japan are collaborating to develop AI-enhanced hospitals, make AI/data science courses mandatory in universities, particularly for healthcare students, and provide online medical AI education resources. This aims to cultivate expertise while leveraging Japan’s universal healthcare system and large volumes of standardized health data to lead in medical AI [145]. In this regard, researchers have made progress in recent years. For example, researchers from Osaka University, JST PRESTO, the University of Tokyo, and RIKEN have developed a deep neural network called “MNet” that can classify multiple neurological diseases using resting-state MEG signals with high specificity. This technology has the potential to improve neurological diagnoses and reduce the burden on clinicians in critical care [146]. In the field of oncology, the University of Tokyo, Shimadzu Corporation, and Juntendo University have developed a predictive model that significantly reduces misclassification rates of diseases compared to using a single tumor marker [147]. Additionally, institutes from Japan, Germany, the US, and Chile have collaborated to enhance the classification of breast tumors using subtle differences in the nuclei of microenvironmental myoepithelial cells [148].

**Figure 4:**
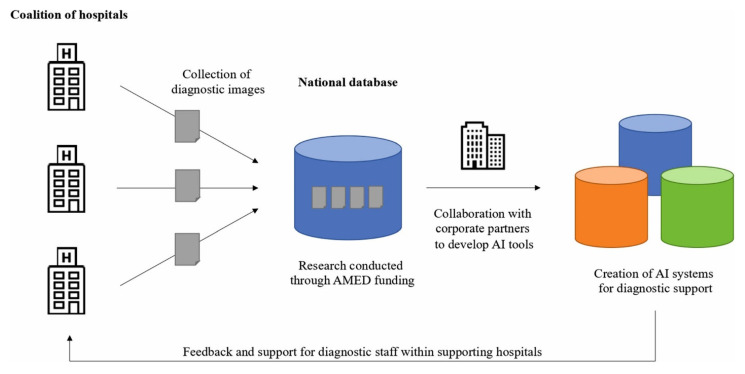
An example of a clinical diagnostic database aimed at promoting the development of supplementary AI tools in healthcare. Reprinted from [145].

It is important to note that regulations require human supervision of AI used for clinical decision support. In addition, challenges such as data privacy, multi-sector collaboration, and developing a robust medical AI workforce should be addressed by bringing together clinicians and data scientists (Figure 4) [145].

## 5. Conclusions and Future Perspective

In conclusion, AI has tremendous potential to revolutionize and accelerate the development of therapies in regenerative medicine. From enhancing drug discovery to optimizing tissue engineering and cellular therapies, AI can provide insights by analyzing vast molecular and genomic datasets that would be impossible for humans to perceive. While AI shows promise to advance regenerative medicine research and development, there are significant technical challenges that must be addressed before these technologies can be widely adopted. One of the significant limitations is the lack of large, high-quality datasets needed to train sophisticated machine-learning models. Regenerative medicine involves complex biological interactions that are difficult to fully capture in data.

Additionally, developing accurate computational models that can simulate and predict cell behavior over time poses immense technical challenges due to our still-limited understanding of cellular and molecular pathways. Validating AI systems and gaining regulatory approval also requires extensive clinical testing, which takes considerable time and resources. Addressing concerns around data privacy, security, and bias and ensuring fair and equitable access to new tools is equally important. Moreover, obtaining clinician buy-in for technologies promising more effective personalized care will require overcoming adoption hurdles. Substantial ongoing research is still needed to overcome these limitations and translate AIs theoretical potential in regenerative medicine into real-world solutions that tangibly improve patient outcomes. Researchers, policymakers, healthcare providers, and AI developers must work together to develop appropriate safeguards, oversight mechanisms, and guidelines for using AI in regenerative medicine. As AI technologies continue to improve and more high-quality data becomes available, the opportunity for refining and customizing AI algorithms specifically for regenerative medicine purposes will increase.

Moving forward, further innovations in areas such as machine learning, natural language processing, computer vision, and robotics have the potential to uncover new insights that could revolutionize how regenerative therapies are developed and delivered. Combining AI with other emerging technologies such as nanotechnology, genome editing, and 3D bioprinting may lead to unprecedented advances in creating personalized regenerative solutions. With the appropriate ethical framework and governance structures in place, the future of AI-driven regenerative medicine seems promising. However, progress will depend on maintaining a human-centric approach that utilizes AI capabilities to serve the best interests of patients and society. Through multidisciplinary collaborations and responsible development and use of these technologies, we may one day realize the full potential of AI to usher in a new era of customized and effective regenerative therapies.

### Summary of the Key Points

- AI can help accelerate drug discovery by analyzing large datasets to identify promising drug candidates and optimize drug properties.
- AI-enabled disease modeling can provide insights into disease mechanisms and aid in the identification of new therapeutic targets.
- AI can improve predictive modeling to identify patients who may benefit from regenerative therapies and optimize treatment plans.
- AI can enable the development of personalized medicine approaches based on a patient’s genetic and health data.
- AI can optimize materials and fabrication methods for tissue engineering applications.
- AI can assist in identifying the most suitable cell types for cell therapies and optimizing cell delivery and monitoring.
- AI can enhance the efficiency and accuracy of clinical trial design.
- AI can be used to monitor patients in real-time to detect changes and risks early.
- AI can provide personalized patient education materials tailored to individual needs and preferences.
- AI can improve regulatory compliance through enhanced data analysis, traceability, and transparency.
- AI also has roles in related fields such as immunotherapy, genetic engineering, nanotechnology, and microfluidics, which can further advance regenerative medicine.

In summary, AI has the potential to significantly enhance various aspects of regenerative medicine research and development through analyzing large and complex datasets, identifying patterns and trends, and making accurate predictions to optimize processes and therapies. However, challenges related to ethics, data quality, and regulation must be addressed to ensure the safe and effective use of AI in regenerative medicine.

## Figures and Tables

**Table 1:** Some of the AI tools and platforms used in drug discovery.

Name	About	References
DeepChem	DeepChem is a Python package that simplifies deep learning in drug discovery, quantum chemistry, and materials science. It offers tools for tasks such as predicting molecular properties and screening for potential drugs. The library includes pre-trained models and datasets to help researchers and developers get started quickly in cheminformatics and computational chemistry.	[35]
DeltaVina	A Python package that uses molecular docking simulations to offer a pre-trained model to help predict and analyze the binding affinities between proteins and ligands. It calculates energy differences between different ligand conformations within a protein binding site, assisting researchers in assessing relative binding affinities. This information is helpful in drug discovery and virtual screening, aiding in selecting potential drug candidates based on predicted binding affinities. DeltaVina utilizes the AutoDock Vina docking program for its calculations.	[33,36]
AlphaFold	A deep learning system developed by DeepMind that predicts the 3D structure of proteins. It uses deep learning algorithms and protein structure databases to accurately determine the folding patterns and spatial arrangements of amino acids in protein sequences. This has significant implications for various scientific fields, including drug discovery and molecular biology. AlphaFold’s exceptional performance in the CASP competition has garnered widespread recognition.	[37,38]
Chemputer	This platform aims to revolutionize the field of chemistry by automating and digitizing the chemical synthesis process. The Chemputer system combines robotics, artificial intelligence, and machine learning to enable the automated design and synthesis of complex molecules. It allows chemists to program and control the synthesis of specific compounds using computer algorithms, reducing the need for manual labor and improving efficiency. The ultimate goal of Chemputer is to accelerate the discovery and development of new chemicals and materials with potential applications in pharmaceuticals, materials science, and other industries.	[39]
Neural graph fingerprint	Neural graph fingerprint is a Python package consisting of a convolutional neural network (deep learning) that operates directly on a graph representation of a chemical compound’s molecular structure. It encodes the structural features and patterns of the compound into a fixed-length vector. This representation is generated by processing the compound’s graph structure and atom features. Neural graph fingerprints are widely used in drug discovery and chemical informatics to analyze large chemical databases, predict compound properties, and assess toxicity. They enable efficient and accurate analysis, aiding in discovering new drug candidates and optimizing chemical properties.	[33,40]
DeepTox	DeepTox is a deep learning-based model that predicts the toxicity of chemical compounds. It uses a combination of molecular fingerprints and deep neural networks (DNN) to analyze the chemical structure and predict the toxicity of a given compound. DeepTox can be used in drug discovery and toxicology research to identify potentially harmful compounds and prioritize safer alternatives.	[41]
AtomNet	AtomNet is a deep learning model developed by researchers at Google. It is specifically designed for drug discovery and pharmaceutical research. AtomNet primarily utilizes convolutional neural networks (CNNs) to analyze chemical structures and predict their properties, such as binding affinity to target proteins. It has been successful in accurately predicting the activity of potential drug candidates, which significantly expedites the drug discovery process.	[42]

**Table 2:** Tissue engineering challenges [22].

Biological Challenges	Engineering Challenges
Selection of suitable cell sources	Selection of biocompatible materials
Providing repeatable cell differentiation conditions	Achieving optimal physicochemical and mechanical properties
Selection of bioactive agents	Developing scaffold fabrication methods

## Data Availability

No data were used for the research described in the article.
